# Overcrowded Cemeteries

**Published:** 1889-09-21

**Authors:** Arthur P. Purey-Cust

**Affiliations:** Chairman of the Council. Deanery, York


### Editor's Letter-Box.

[Our Correspondents are reminded that prolixity is a great bar to publication, and that brevity of style and conciseness of statement greatly facilitate early insertion.]

### OVERCROWDED CEMETERIES.

In view of the conferences on burial reform to be held during the Church Congress in Cardiff, on September 30; in the Council-chamber, Oxford, on October 8; at the Church-house, Westminster, on October 10; in the Council-chamber, Cambridge, on October 15; and at Armagh, Belfast, Londonderry, Cork, and elsewhere, kindly permit me to point out that the Burial Service in our Book of Common Prayer offers suggestions of a mode of disposing of the dead which has been declared by scientific men to be in accordance with sound science and sanitary law, and which, if properly and completely carried out, renders overcrowding impossible. The rubric, "The priest and clerks meeting the corpse, and going before it either into the church or towards the grave," permits the body, when there is danger of infection, to be taken direct to its burial, instead of into the church. The rubric, "While the body is made ready to be laid into the earth," points to an interment of the body in as close contact with the earth as circumstances, decency, and reverence permit. Another rubric—"While the earth is being cast upon the body by some standing by"—seems to imply that the body is to be surrounded and covered with sufficiency of earth. The mode of burial indicated by these rubrics in the "Order for the Burial of the Dead," in the Prayer Book, is harmless to the living. If the coffin be of a perishable nature, if the soil be dry and porous, if the graves be not too crowded, the dead are resolved into air and ashes in from three to seven years, and this without injury to the living. Now, if the Burial Service manifestly enjoins a mode of disposing of the dead which is in conformity with sanitary laws, it may be inferred that all other supplementary, or exceptionally necessary, sanitary precautions are to be welcomed, and acted upon. Mourners are not expected to do anything, or leave anything undone, to the imperilling of their own welfare, or that of the public at large. It should be considered a pious duty to bury as soon after death as signs of dissolution appear. The coffin should be of some readily perishable material. If the presence of infectious germs be suspected, some chemical compound, capable of destroying such germs, should be placed in the coffin as soon after death as possible. The placing of the body in a properly appointed mortuary near the burying-place should be considered to show as much respect as following it with a costly procession through the crowded streets. The disease-carrying pall should be discarded. The grave should be so shallow as that the air be not excluded. When the soil of the cemetery is not suitable for the disintegration of human remains, the grave should be filled up with dry, porous, properly prepared earth. Bricked graves and vaults, which retain the body in a state of arrested decomposition, should be abandoned. The surface of the grave should not be covered with slabs or monuments preventing the growth of plants and excluding the air. Suitable vegetation should abound. Only when assured of the complete dissolution and redistribution of the first, should a second body be interred in the same earth. Graveyards should be gardens where the dead are buried side by side, each succession of human bodies passing away into air and ashes, the earth being thus ready every succeeding generation to perform its beneficent action again. Thus, natural laws will have been observed, and the earth, which is the best deodoriser and antiseptic known, and the receptacle of all creatures which have lived and died, will have acted as the medium through which the air descends and performs its purifying and disintegrating action, to reascend in new combinations, and nourish fresh life. I crave, therefore, the moral and practical support of your readers on behalf of the Church of England Burial, Funeral, and Mourning Reform Association, whose aim is to abolish the prevalent, improper, imperfect, falsely so called burial in durable coffins, in vaults, or already crowded graves; and to substitute the Church's "earth to earth" mode of burial, in a readily perishable coffin of compressed pulp, or the like, as carried out by the Necropolis Company at the Woking Cemetery. Under this system, not only is the natural chemical combustion of the body brought about, with harm to none, and overcrowding made impossible, but also other distinct and definite advantages accrue; the funeral ceremonies are simplified, the expense lessened, and the same earth rendered available for the burial of the dead, generation after generation, for all time to come.

Arthur P. Purey-Cust, D.D.,

Chairman of the Council.

Deanery, York, Sept. 13th, 1889.

